# Supplementary material for: Content-rich biological network constructed by mining PubMed abstracts
Source: BMC Bioinformatics. 2004 Oct 8;5:147. doi: 10.1186/1471-2105-5-147 (PMC528731; doi:10.1186/1471-2105-5-147)
Supplement: Additional File 5 — The original Chilibot query results of the term "long-term potentiation (LTP)" and 22 other terms, limiting the latest references analyzed to the years 1990, 1995, 2000, and 2004. [file 1471-2105-5-147-S5.bz2 › chilibotAdditionalFile5/ltp2004/html/TRKA_PKC.html]

 


 **TRKA** and **PKC** 
  
Found 28 abstracts in PubMed,  **28 abstracts were retrieved and analyzed**.  


---

 Search Google  |
 PDF files only 
|  EDU domain only 

---

**Interactive relationship** (e.g. stimulation, inhibition, etc)

- In this report, we review the possible roles of the intracellular proteins that directly interact with the p75 NTR, atypical protein kinase C  **PKC**  binding protein, p62 and second messengers in the functional antagonism exhibited by  **TrkA**  and p75 NTR with an emphasis on the nuclear factor kappa B activation pathway.  Ref: 11992464 J Neurosci Res, 2002
- Analysis of phosphorylation of the endogenous  **PKC**  substrate myristoylated alanine rich C kinase substrate MARCKS, and of subcellular distribution of  **PKC**  alpha, delta, and epsilon revealed that NGF only caused a very small activation of  **PKC**  in SH SY5Y  **TrkA**  cells.  Ref: 11257453 Cell Signal, 2001
- In the present work, specific activation of protein kinase C  **PKC**  isoforms and of intracellular signal transduction pathways, which include nerve growth factor NGF receptor  **trkA** , phospholipase C PLC gamma 1 and extracellular regulated kinases ERKs 1 and 2, by the recombinant C terminal portion of the TeTx heavy chain H C TeTx is reported.  Ref: 11336640 Biochem J, 2001
- The less effectivity of NGF via  **TrkA** , which was immunocytochemically shown in human OL, and of TPA via the protein kinase C  **PKC**  pathway, may have its common focus on the mitogen activated protein kinase MAPK cascade.  Ref: 11276121 Microsc Res Tech, 2001
- The human neuroblastoma cell line SH SY5Y  **TrkA**  differentiates in vitro and acquires a sympathetic phenotype in response to phorbolester activator of protein kinase C,  **PKC**  in the presence of serum or growth factors, or nerve growth factor NGF.  Ref: 11257453 Cell Signal, 2001
- Tyrosine phosphorylation and activation of  **PKC**  iota were inhibited in a dose dependent manner by both PP2 and K252a, src and  **TrkA**  kinase inhibitors.  Ref: 11713277 Mol Cell Biol, 2001

**Parallel relationship** (e.g. studied together, co-existance, homology, etc.)

- This increase was inhibited by K 252a, a trk A  [ **TRKA** ]  receptor inhibitor, and calphostin C, a  **PKC**  inhibitor.  Ref: 11406293 Brain Res Mol Brain Res, 2001
- Furthermore, we demonstrate activation of src by NGF along with formation of a signal complex including the  **TrkA**  receptor, src, and  **PKC**  iota.  Ref: 11713277 Mol Cell Biol, 2001
- We have now investigated to what extent phorbolester and NGF cause activation of Ras and Raf 1 and the involvement of  **PKC**  in this response in differentiating SH SY5Y  **TrkA**  cells.  Ref: 11257453 Cell Signal, 2001
- A series of disubstituted alkylthio methyl and alkoxymethyl K 252a derivatives was synthesized with the aim of enhancing and separating the neurotrophic properties from the undesirable NGF trk A  [ **TRKA** ]  kinase and  **PKC**  inhibitory activities of K 252a.  Ref: 9191963 J Med Chem, 1997
- Activation of signal transduction pathways involving  **trkA** , PLCgamma 1,  **PKC**  isoforms and ERK 1 2 by tetanus toxin.  Ref: 10996319 FEBS Lett, 2000
- In the present work the differential activation of  **PKC**  isoforms and of signal transduction pathways, including nerve growth factor receptor  **trkA** , phospholipase Cgamma 1 PLCgamma 1, and extracellular regulated kinases 1 and 2 ERK 1 2 by TeTx in a synaptosome enriched P 2 fraction from rat brain is reported.  Ref: 10996319 FEBS Lett, 2000
- Compound 21 exhibited good selectivity against numerous tyrosine and serine threonine kinases including  **PKC** , Tie2,  **TrkA** , CDK1, p38, JNK, and IRK.  Ref: 14640546 J Med Chem, 2003
